# Partial characterization of starches from major banana (matooke) cultivars grown in Uganda

**DOI:** 10.1002/fsn3.505

**Published:** 2017-08-31

**Authors:** Umar L. Ssonko, Florence I. Muranga

**Affiliations:** ^1^ Department of Food Science & Technology Makerere University Kampala Uganda; ^2^ Presidential Initiative on Banana Industrial development (PIBID) Kampala Uganda

**Keywords:** Banana starch, East African Highland banana, Extraction, Granule size, Pasting properties

## Abstract

The SEM of starch from five EAHB cultivars showed a mixture of irregular granule shapes with smooth surfaces. The starches' average diameter size ranged between 16.31 and 21.98 μm. The moisture, protein, and ash content of the starches were: 11.12%–11.84%, 0.1% and 0.23%–0.47% respectively. The amylose content of these starches was between 11% and 13%. The starches peak viscosity ranged between 488.42 and 558.66 RVU. The EAHB starches exhibited relatively low pasting temperatures (<75°C), a high peak viscosity (488.42–558.71 RVU), high level of viscosity breakdown (235.00–311.92 RVU) and low set‐back values (61.21–104.33 RVU). In general, the EAHB starches' WHCs and SPs increased substantially at 80°C with maximum SP (12.43–14.27 g water/g starch) and solubility (12.52%–14.19%) values obtained at 90°C. The starch clarity ranged from 1.7% to 2.3% and followed the same pattern. The starches exhibited poor freeze‐thaw stability as they had high syneresis (68% to 72%) after 10 freeze‐thaw cycles.

## 1 INTRODUCTION

Starch is a polymer of glucose, which serves as the main energy reserve in higher plants. The main botanical sources of starch are wheat, maize, potato, and cassava with only minor quantities from rice and other starches produced (Waterschoot, Gomand, Fierens, & Delcour, 2015). Starch is an inexpensive, abundant, biodegradable and renewable material that is available from a variety of botanical sources. Since the end of the 20th century, an assortment of applications for starch has been discovered with respect to food and non‐food industries (Bello‐Pérez & Paredes‐López, 2009). Due to broadening of the spectrum of starch applications as well as the desire by development agencies to promote regional economies and sustainable agricultural activities, use of non‐traditional starch botanical sources, such as roots and tubers (Hoover, 2001), tropical fruits (Zhang, Whistler, BeMiller, & Hamaker, 2005), among others has been sought in recent decades.

Kayisu, Hood, and Vansoest (1981) having isolated and characterized starch from green bananas, subsequent studies on banana starch raised interest with regard to carrying out more investigations in its properties as well as its suitable applications as results revealed that banana starch had peculiar physicochemical, functional, and digestibility features (Zhang & Hamaker, 2012) in comparison with the traditional starch sources (BeMiller, 2007). Bananas are mainly grown in the tropical and sub‐tropical regions of the world with an estimated annual world production of about 105 million MT in 2012 according to FAO.

Banana production for export is considered a different economic and technological activity to banana production as a staple. Production for export relies on only a few varieties, which were selected for their high yields, durability in long distance transport, consistent quality and unblemished appearance. Likewise, EAHB production for industrial raw material as a starch source requires relying on a few selected cultivars based on high yields especially in terms of starch content and consistent quality in terms of physicochemical starch properties.

Studies have revealed that the physicochemical and functional properties of banana starch are dependent on variety, regional climatic conditions, and harvesting periods (Bello‐Perez, Agama‐Acevedo, Sanchez‐Hernandez, & Paredes‐Lopez, 1999). An assortment of different banana varieties and cultivars are produced at regional levels despite the Criollo variety being the most commercialized banana cultivar worldwide. For instance, the East African highland bananas (EAHB)—AAA cultivars are commonly cultivated and are a staple food for the Great Lakes region of East Africa (Gold, Bagabe, & Ssendege, 1999) and in particular Uganda where they are a key component in both food security and agricultural sustainability (Tenywa, Isabirye, Lal, Lufafa, & Achan, 1999).

An array of cultivars of the EAHB is extensively produced and consumed in many parts of Uganda, where some cultivars are more preferred than others mainly due to the cooking and tasting characteristics. The EAHB is mainly consumed when green during which stage starch is the main component (≥70% Dry Weight Basis (DWB) (Muranga, 1998). Due to the bananas' perishability leading to high postharvest losses and low value to bulk ratio, production of banana flour from the EAHB has been sought in Uganda and neighboring countries.

Although, banana starch characterization has been carried out extensively in the last two decades (Bello‐Perez et al., 1999; Eggleston, Swennen, & Akoni, 1992; Muranga, 1998; Nwokocha & Williams, 2009; Waliszewski, Aparicio, Bello, & Monroy, 2003; Zhang & Hamaker, 2012), some characterization of starch from a few EAHB cultivars has been done by only one researcher (Muranga, 1998) yet the botanical source of starch is determinant of the chemical composition, granule morphology, amylose:amylopectin ratio and arrangement of the macro molecules, which in turn determine the physicochemical and functional properties of the starch. Since the EAHB are set to transform from being a regional food staple to a crop with numerous industrial applications, the characterization of starch from EAHB cultivars was considered.

Consequently, studies on the properties of starches from regional banana cultivars have increasingly become important due to the possibility of diversifying their applications, which would transform these bananas into a commercially viable commodity (Zhang et al., 2005). The examination of the rheological and morphological properties of starches from bananas is of particular importance in the characterization and understanding of their functional properties.

Thus, the aim of this study was to characterize the chemical composition, average particle size, morphology, swelling, solubility, pasting properties, paste clarity, and freeze thaw stability of starch isolated from the Enyeru, Mbwazirume, Nandigobe, Mpologoma, and Bukumu East African highland banana cultivars from Western Uganda which represent the three sub groups for the EAHB available and also are among the most popular cultivars. The results of this study are expected to provide useful technical information about the physicochemical and functional properties of these starches, which will provide a foundation for enhancing their application in the food industry in substitution of commercially available starches.

## 2 MATERIALS AND METHODS

### 2.1 Materials

Five cultivars of edible green (unripe) pre‐climateric EAHB (*Musa accuminata* AAA‐ EA) namely: Nandigobe, Enyeru, Mpologoma, Mbwazirume, and Bukumu were selected from a 24‐acre demonstration plantation of the Presidential initiative on Banana Industrial Development (PIBID) project in Bushenyi district, which is at an altitude of about 1607 m above sea level with bimodal rainfall seasons receiving an average rainfall of about 1233 mm and average temperatures of 19.3°C. The plants were cultivated under normal conditions of cultivation.

### 2.2 Starch Isolation

Starch was prepared by a modified method of Muranga (1998). In brief, the bananas were peeled, sliced, and crushed in a blender after holding in NaOH solution for 10 min. The slurry was diluted with water, strained over 500 μm sieves and the filtrate held at 4°C. After 6 hr, the starch was washed repeatedly and left to dry at room temperature for 72 hr. The dried starch was milled, passed through a 200 μm sieve and stored in air tight glass containers till further analyses.

### 2.3 Chemical composition

The moisture, ash, fat, and crude protein were determined by standard procedures (AOAC, 1995). Purity of isolated starch was determined polarimetrically using an Automatic polarimeter (Atago, AP—300). Amylose content was determined according to Williams et al.*'*s Williams, Kuzina, and Hlynka (1970) method. Ten mL of 0.5 N KOH were added to 20 mg of starch samples, mixed thoroughly and topped up in 100 ml volumetric flask with distilled water. Ten ml aliquots were pipetted in 50 ml volumetric flasks to which 5 ml of 0.1 N HCl and 0.5 ml of iodine reagent were added sequentially. The flasks were diluted to the mark and absorbance was measured at 625 nm. An amylose and amylopectin blend standard curve was used to measure the amylose content.

### 2.4 Starch granule morphology

The granule micrographs were obtained using a JSM 6060 model SEM (JEOL Co., Ltd, Japan). The EAHB starch powders were placed on the SEM stubs using a two–sided adhesive tape (Nisshin EM Co., Ltd). The specimen stub was subsequently coated with Pt‐Pd using an MSP‐1S magnetron sputter coater (Vacuum Device Inc.). The coated samples were then analyzed using the SEM operated at 10.0 KV.

### 2.5 Particle size analyses

Particle size was analyzed with a Laser scattering particle size analyzer (SALD‐7100, Shimadzu Corporation) installed with a batch sample cell using methyl propanol as the solvent. The percentages of the number of granules corresponding to the different sizes were recorded and average granule diameter was obtained directly using the equipment's software.

### 2.6 Pasting properties

The pasting properties of the EAHB starch samples were measured by a Rapid ViscoAnalyser (RVA‐4, 1998, Newport Scientific Pty. Ltd, Australia). The General pasting method STD1 profile (ICC, 1995) was used for all the samples with a modification of holding temperature to 90°C instead of 95°C due to the high altitude of our laboratory location. Data were directly calculated from the pasting curve, using Thermocline for Windows v3.0 (TCW3) software for the RVA.

### 2.7 Swelling power and solubility

These were determined as described by Waliszewski et al. (2003). Starch suspensions (1% w/w) were heated to 50, 60, 70, 80, and 90°C for 30 min with 5 min intermittent shaking. The slurry was centrifuged for 15 min at 3,000*g* using a bench top centrifuge (Eppendorf, 5810). The supernatant was decanted and the volume determined. The sediment was dried in an oven for 2 hr at 110°C and the swelling power (SP) determined by difference.

### 2.8 Paste clarity

Paste clarity was determined according to the method by Waliszewski et al. (2003). In brief, 4% starch suspension in a screw cap tube was heated in boiling water with vigourous shaking at 5 min intervals. After 30 min, the samples were cooled to room temperature and refrigerated to 6°C for 72 hr. The percentage transmittance at 650 nm was determined every 24 hr against a water blank in a Cary 100 UV—Vis spectrophotometer, (Agilent Technologies).

### 2.9 Freeze–thaw stability

This was evaluated using Bello‐Perez et al.'s (1999) method. Briefly, 5 ml of starches suspensions (5% w/v) were rapidly heated to 75°C in a water bath subjected to constant agitation. After a 30 min hold time, the gels were cooled and stored at 20°C, and after 18 hr, they were thawed to 28°C for 6 hr. The water exuded by the gels was determined gravimetrically by vortexing the thawed gels for 15 s followed by centrifugation at 1620g for 10 min. The amount of water exuded after each freeze–thaw cycle was measured and expressed as a percentage of water separated. Ten freeze–thaw cycles were performed in total.

## 3 RESULTS AND DISCUSSION

### 3.1 Chemical composition

The chemical composition of the starches from the different EAHB cultivars is presented in Table 1.

**Table 1:** Chemical analysis of the EAHB starch from five cultivars

Starch origin	Starch DB (%)	Amylose (%)	Moisture (%)	Ash (%)	Protein (%)	Lipids (%)
Mbwazirume	99.6^a^	11.96^a^	11.47 ± 0.18^a^	0.37^a^	0.1^a^	ND
Mpologoma	99.4^b^	12.13^b^	11.84 ± 0.15^b^	0.47^b^	0.1^a^	ND
Enyeru	99.5^a,b^	12.83^c^	11.36 ± 0.17^a^	0.37^a^	0.1^a^	ND
Bukumu	99.7^a^	12.74^d^	11.12 ± 0.13^a^	0.24^c^	0.1^a^	ND
Nandigobe	99.6^a^	12.59^e^	11.18 ± 0.14^a^	0.23^c^	0.1^a^	ND

ND, Not detected.

Values are means (*n* ≥ 3). Values with the same superscript letter are not statistically significant at the 5% level.

The moisture content (MC) of the starch from the five EAHB cultivars ranged from 11.1% to 11.8% and protein content was 0.1% (Table 1), which were close to the results reported by Kayisu et al. (1981) of 10.8% MC and 0.2% protein content. Muranga (1998) reported the MC for starch from EAHB to range from 9.7% to 14.4% and protein content to be 0.1%. Lipids were not detected by the usual methodologies used to analyze proximate composition of foods (AOAC, 1995). This unusual low content of oil in the starch from the EAHB cultivars presents potential technological use of the starch in many industrial applications as challenges arising from rancidity of the oil in the starch are not expected to arise in the EAHB starch. The ash content (0.23%–0.47%) was higher than that reported by Kayisu et al. (1981) of 0.02% and Muranga, 1998) of 0.1%–0.2% but was in close range with that reported by Eggleston et al. (1992) for plantains (0.27%–0.34%), plantain hybrids (0.28%–0.32%) and cooking bananas (0.35%–0.41%). The high ash content may be attributed to the high mineral content of the banana starch especially potassium rather than impurities as the starch obtained was over 99% pure. Bello‐Pérez, de Léon, Agama‐Acevedo, and Paredes‐López (1998) reported higher results of protein (2.03%), fat (2.46%) and ash (0.54%) for banana.

The amylose content of the starches from the five EAHB cultivars was in the range of 11.96–12.83% (Table 1). Though amylose is a smaller fraction in most starches, it has a large influence on the starch properties due to their structural contribution to the amorphous component of the starch granules. Different researchers have reported varying amylose contents of banana starch: 16% (Kayisu et al., 1981); ~17% for Cavendish (Garcia & Lajolo, 1988); 40.7% for Valery (Waliszewski et al., 2003); 10%–11% for plantains (Eggleston et al., 1992); 38.6%–43.8% for six Kluai cultivars (Vatanasuchart, Niyomwit, & Wongkrajang, 2012). Muranga (1998) reported amylose content of 8.44–12.94 for starch from EAHB. Relatively high amylose content of 12.59% was obtained for Nandigobe cultivar in comparison to 8.44% reported for the same cultivar by Muranga (1998). This difference may be due to the high altitude at which the bananas used in this study were grown as well as other environmental conditions. Variable amylose contents have been reported for different starch sources: corn starch (30.0%) (Bello‐Pérez et al., 2006); rice starch (28.6%) (Wani et al., 2013), cassava starch (13.6% and 27.0%), and potato starch (8.5% and 38%) (Moorthy, 2004).

### 3.2 Granule characteristics

**Figure 1:**
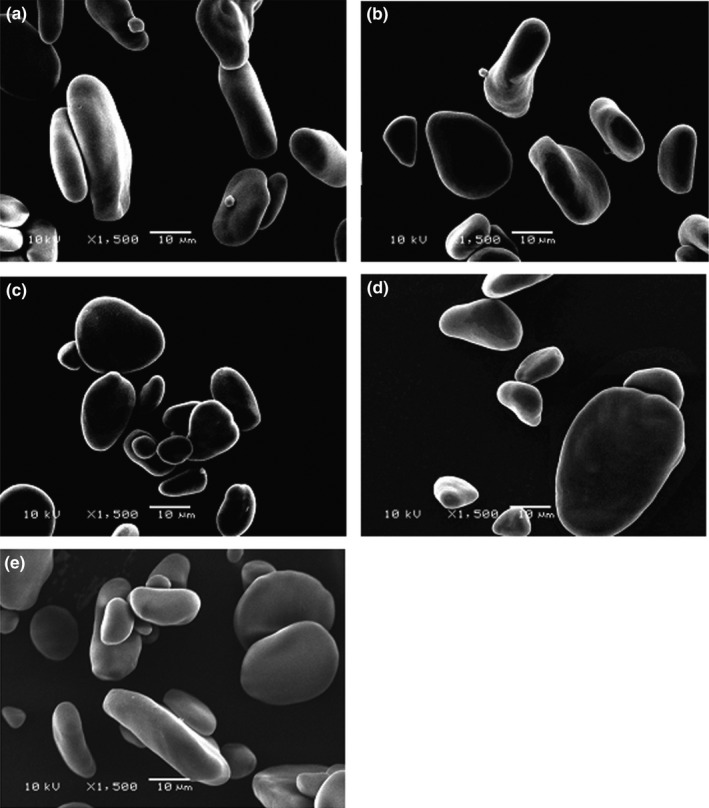
Scan electron micrographs for banana starch from 5 EAHB cultivars ((a) Mbwazirume, (b) Nandigobe, (c) Mpologoma, (d) Bukumu, (e) Enyeru)

Figure 1(a–e) shows the scan electron micrographs of starch from the five EAHB cultivars.

The EAHB starches showed a mixture of irregular granule shapes of elongated, spheroid and oval granule shapes with smooth surfaces devoid of dents (Figure 1). These results were in agreement with those of other researchers who reported that banana starch was composed of smooth irregularly shaped granules with different shapes (Kayisu et al., 1981; Muranga, 1998). Waliszewski et al. (2003) also reported a granule shape for Valery banana starches similar to that obtained in this study. Eggleston et al. (1992) reported starch from Fougamou, a cooking banana variety to have granules that are predominantly triangular and pear shaped. Smooth irregular shaped bimodal granules were found to constitute white plantain starch (Nwokocha & Williams, 2009). The absence of surface dents in the EAHB starch granules may provide low starch reactivity when subjected to chemical treatment since the presence of dents on corn starch granules was reported to affect corn starch reactivity during chemical treatment in addition to affecting its functional and physicochemical properties (Huber & BeMiller, 2001).

### 3.3 Granule size

The average diameter size for the starches from the five EAHB cultivars was 16.31–21.98 μm (Table 2). The starch average diameter size for the different cultivars were significantly different from each other except for Nandigoge and Bukumu. Muranga (1998) reported the average banana starch granule size to range from 19.67 to 30.06 μm. The study by Eggleston et al. (1992) revealed that the average diameter for starches from plantain, plantain hybrids and cooking bananas to be in the ranges 24.1–26.6 μm, 26.4–35.1 μm and 16.4–30.9 μm respectively. Nunez‐Santiago, Bello‐Perez, and Tecante (2004) found starch from banana (*Musa paradisiaca*) to have an average diameter of 24.31 μm. Waliszewski et al. (2003) reported that the granule size for Valery banana starches was 14–88 μm in width and 21–108 μm in length, which are larger dimensions. Coulibaly, Nemlin, and Amani (2006) reported that the average granule sizes for Hybrid CRBP 14, hybrid CRBP 39, hybrid FHIA 17, hybrid FHIA 21 and banana variety Orishele were 20.1 ± 3.9 μm, 17.9 ± 3.7 μm, 19.0 ± 4.3 μm, 18.8 ± 4.6 μm, and 18.8 ± 4.6 μm respectively.

**Table 2:** Particle size of EAHB starch from five cultivars

Banana starch	Average particle size (μm)
Mbwazirume	21.98 ± 0.17^a^
Nandigobe	18.70 ± 0.27^b^
Mpologoma	16.31 ± 0.34^c^
Bukumu	18.59 ± 0.22^b^
Enyeru	19.89 ± 0.03^d^

Values are means (*n* = 3). Values with the same superscript letter are not statistically significant at the 5% level, LSD = 0.4222 (*p* = .05).

### 3.4 Swelling Power

The SP results of the five EAHB cultivar starches are given in Table 3. At <70°C, SP values were minimal (<1 g water/g starch) and there was no significant difference in the SP values registered for all the five EAHB cultivar starches (Table 3). Minimal SP values are indicative of the stability of starch granules in this temperature range, which may be attributed to being temperatures lower than the gelatinization temperatures for the starches from the different EAHB cultivars. The greatest increase in SP was observed from 70°C to 80°C for all the starches with SP values of 8.87, 7.48, 7.68, 7.75, and 8.95 g water/g starch for Mbwazirume, Mpologoma, Enyeru, Bukumu, and Nandigobe starch respectively with significant difference between SP values between some of the cultivars at 70°C, 80°C and 90°C. The SP results at temperatures higher than 70°C were in agreement with data by de la Torre‐Gutiérrez, Chel‐Guerrero, and Betancur‐Ancona (2008). At >70°C, the starches swelled rapidly due to the breaking of intermolecular hydrogen bonds in amorphous areas, which allows irreversible and progressive water absorption, the same pattern reported by Bello‐Perez et al. (1999) for “macho” banana starch. The maximum SP for all the starches were obtained at 90°C (12.43–14.27 g water/g starch) which were lower than that for starch from “macho” variety of banana (31.1 g water/g starch) (Bello‐Pérez et al., 1998), yellow and white plantain starch (~17 g water/g starch) (Nwokocha & Williams, 2009), corn starch (16 g water/g starch) (Anggraini, Sudarmonowati, Hartati, Suurs, & Visser, 2009), cassava starch (40–50 g water/g starch) (Anggraini et al., 2009), and amaranth starch (20 g water/g starch) (Bello‐Pérez et al., 1998).

**Table 3:** SP of native starch from five EAHB cultivars

Sample	50°C (g/g)	60°C (g/g)	70°C (g/g)	80°C (g/g)	90°C (g/g)
Mbwazirume	0.69^a^	0.86^a^	2.45^a^	8.87^a^	12.61^a^
Mpologoma	0.72^a^	0.79^a^	2.84^b^	7.48^b^	12.43^a^
Enyeru	0.70^a^	0.85^a^	3.28^c^	7.68^b^	14.27^b^
Bukumu	0.74^a^	0.82^a^	2.93^b^	7.75^b,c^	13.98^c^
Nandigobe	0.76^a^	0.78^a^	3.89^d^	8.95^a^	14.10^b^

g/g, g of water retention g^−1^ of starch.

Values are means (*n* = 3). Values with the same superscript letter are not statistically significant at the 5% level. LSD = 0.2128 (*p* = .05).

### 3.5 Solubility

Table 4 presents the starch solubility results for the five EAHB cultivars as a function of temperature between 50°C and 90°C.

**Table 4:** Starch solubility with respect to temperature carried out using 1% db (w/w) starch dispersions of the five EAHB cultivars (the starch was heated from 50°C to 90°C for 30 min

Starch source	50°C (%)	60°C (%)	70°C (%)	80°C (%)	90°C (%)
Mbwazirume	0.65^a^	0.85^a^	2.48^a^	8.80^a^	12.80^a^
Mpologoma	0.68^a^	0.80^a^	3.80^b^	8.90^a^	12.52^a^
Enyeru	0.69^a^	0.85^a^	3.26^c^	7.67^b^	14.14^b^
Bukumu	0.74^a^	0.80^a^	2.90^d^	7.92^b^	13.90^b^
Nandigobe	0.65^a^	0.82^a^	2.88^c^	7.53^b,c^	14.19^b^

Values are means (*n* = 3). Values with the same superscript letter are not statistically significant at the 5% level. LSD = 0.0.3435 (*p* = .05).

The starch solubility of the EAHB cultivars varied from 0.65% to 14.19% in the 50°C to 90°C temperature range (Table 4). At ≤70°C, the starch solubility increased only slightly (2.48%–3.8%), however, at 80°C the starch solubility of all the EAHB cultivars increased considerably (7.53%–8.9%). The maximum starch solubility (12.52%–14.19%) for all cultivars was observed at 90°C and at temperatures beyond or equal to 70°C significant differences were observed in the Swelling power of starches from the different cultivars as indicated in Table 4..

Nandigobe, Enyeru, and Bukumu starches exhibited the highest solubility at 90°C (14.19%, 14.14% and 13.9% respectively) with no significant differences, while Mpologoma and Mbwazirume starches (12.52% and 12.8% respectively) exhibited lower solubility values that were not significantly different from each other at 90°C.

The starch solubility for the EAHB cultivars was lower than that reported by Coulibaly et al. (2006) for native starches from banana hybrids, plantain hybrids and plantain variety Orishele in the temperatures between 65°C and 95°C, which were; 0.93%–21.70% for hybrid CRBP 14, 1.15%–28.20% for hybrid CRBP 39, 1.63%–14.06% for hybrid FHIA 17, 1.63%–14.06% for hybrid FHIA 21 and 0.95%–21.06% for the plantain variety Orishele. Nwokocha and Williams (2009) established that the solubility values for white and yellow plantain starches in the temperature between 60°C and 90°C was about 0.2%–6.8%. The general solubility pattern of the EAHB cultivar starches were very similar to their swelling patterns as the highest increase in solubility and SP were recorded at 80°C and minimal and maximum solubility and SP values obtained at ≤70°C and 90°C respectively.

### 3.6 Banana starch pasting profile

The pasting of the EAHB starches exhibited gradual viscosity increase with increasing temperature during the heating stage (50°C–90°C) until peak viscosity was attained (Table 5). The peak viscosity of the cultivars of the EAHB starches investigated was between 488.42 and 558. 71 RVUs. During the holding step at 90°C, the viscosity decreased due to molecular dissociation. The EAHB starches exhibited high pasting temperatures, a high peak viscosity, high level of viscosity breakdown and lower set‐back values (Table 5). The difference in pasting temperatures between the EAHB cultivars may be due to the differences in the bonding forces between starch chains, whereby the higher pasting temperature indicates stronger bonding forces and thus requires elevated temperatures to overcome these forces.

**Table 5:** Pasting properties of starches isolated from the EAHB cultivars

Sample	Pasting time (min)	Pasting temperature (°C)	Peak viscosity (RVU)	Minimum viscosity (RVU)	Final viscosity (RVU)	Breakdown viscosity (RVU)	Setback viscosity (RVU)
Mbwazirume	4.67^a^	74.50^a^	492.08^a^	215.08^a^	319.42^a^	277.00^a^	104.33^a^
Mpologoma	4.60^b^	73.05^b^	488.42^a^	242.71^b^	314.13^b^	245.71^b^	71.42^b^
Enyeru	4.64^c^	74.48^a^	510.79^b^	221.88^c^	325.33^c^	288.92^c^	103.46^a^
Bukumu	4.62^d^	73.90^c^	520.08^c^	285.08^d^	363.33^d^	235.00^d^	78.25^c^
Nandigobe	4.44^e^	70.20^d^	558.71^d^	246.79^e^	308.00^e^	311.92^e^	61.21^d^

Values are means (*n* = 3). Values with the same superscript letter are not statistically significant at the 5% level.

The increase in viscosity during the heating cycle is influenced by the extent of amylose leaching, granular swelling and the extent of friction between swollen granules. The higher peak viscosity displayed by Nandigobe could be attributed to its greater SP (Table 3) and relatively large granule size (Table 1). Srichuwong, Sunarti, Mishima, Isono, and Hisamatsu (2005) have postulated that starches with larger granules might occupy more volume and thus enhance viscosity. The pasting properties of starches from the different cultivars of the EAHB were significantly different. However, from the application point of view these differences were marginal and would not affect the application suitability with respect to their pasting properties of interchanging the cultivars.

It is difficult to compare the RVA results obtained in this study with those reported for other starches, due to differences in starch concentration and to the methodology (Brabender viscoamylogram, micro viscoanalyzer) used for determination of their pasting characteristics. However, EAHB starch exhibited lower temperature (<75°C) for paste development in comparison to corn starch (>80°C) (Bello‐Pérez et al., 2006) and higher viscosity (>483.33 RVUs) in comparison to corn starch (~500 BU) (Bello‐Pérez et al., 2006), which is an interesting feature for products that require high viscosity at lower processing temperature, and also an advantage for the development of new products with thermolabile ingredients.

### 3.7 Paste clarity

**Figure 2:**
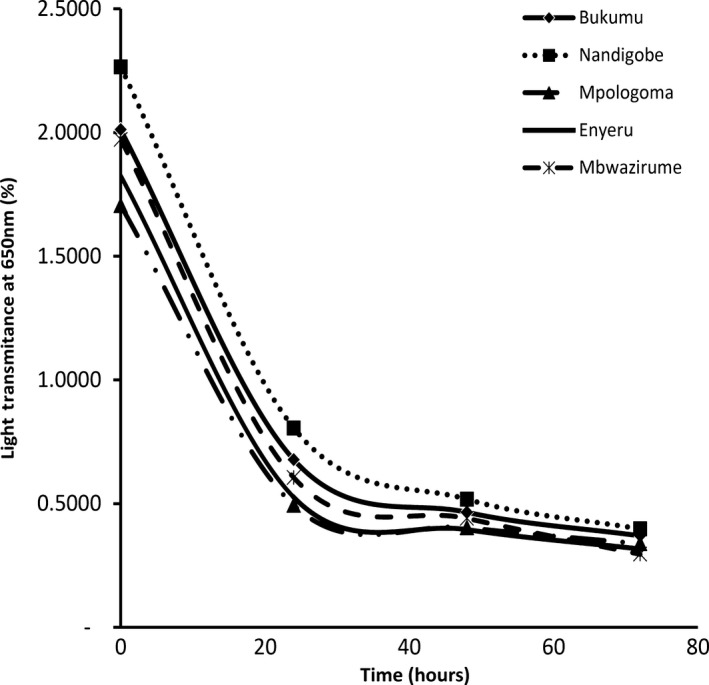
Paste clarity for starch from five EAHB cultivars (Bukumu, Nandigobe, Mpologoma, Enyeru and Mbawzirume)

The starches for all the banana cultivars followed the same trend. The starch clarity (i.e. transmittance value) of the starches from the EAHB cultivars ranged from 1.7% to 2.3% (Figure 2). Results showed these starches to be far less translucent than white and yellow plantain starch at the same concentration (~6% and 7.5% respectively) (Nwokocha & Williams, 2009). Hoover, Sailaja, and Sosulski (1996) reported that degree of transmittance is directly affected by degree of water absorption capacity. This phenomenon is in agreement with the EAHB cultivar starch results whereby Nandigobe, which had the highest WHC furthermore, had the highest paste clarity while Mpologoma starch, which had the lowest WHC similarly, had the lowest paste clarity. Comparison of the paste clarity results between the EAHB cultivar starches and most starches from other sources was not feasible due to the differences in methods and concentrations used by the various researchers. Since paste clarity is a quality characteristic due to its contribution of cloudiness and polish to product colour, EAHB starch has the potential for use in food applications that require low transparency, such as puddings, sauces and dressings for salads and seasonings.

### 3.8 Freeze–thaw stability

**Figure 3:**
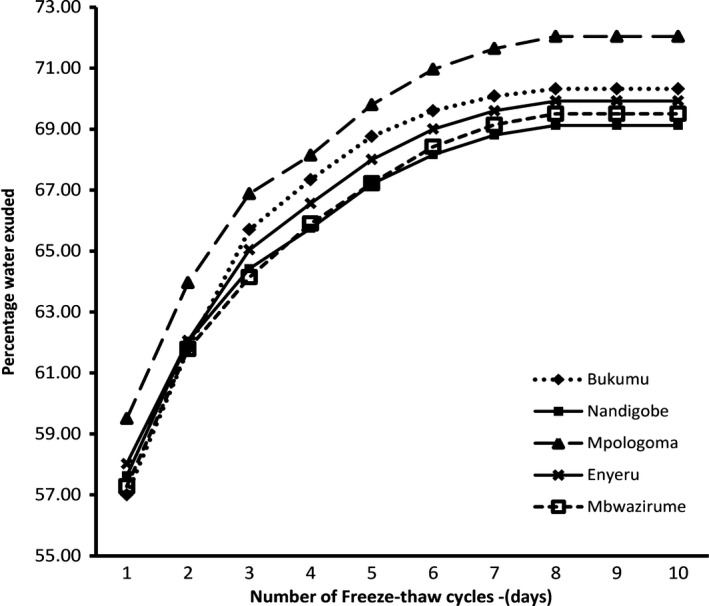
Freeze thaw stability for starch from five EAHB cultivars (Bukumu, Nandigobe, Mpologoma, Enyeru and Mbawzirume)

Freeze–thaw stability is one of the quality characteristic of starch gels. When a starch gel is subjected to repeated freezing and thawing cycles, it releases water a condition termed as syneresis. The extent of syneresis is a measure for its freeze–thaw stability (Goff, 2004).The starch from the cultivars of the EAHB exhibited poor freeze‐thaw stability (Figure 3). All the EAHB cultivar starches had high syneresis (>55%) after the first freeze‐thaw cycle, which indicates poor freeze‐thaw stability. However, the same starches did not undergo much syneresis during the subsequent freeze‐thaw cycles and after 10 freeze‐thaw cycles, the total syneresis was between 68% and 72% for the EAHB starches.

Mpologoma starch, which had the smallest mean granule diameter and lowest SP, exhibited the lowest freeze–thaw stability while Mbwazirume and Nandigobe starch starches, which had the biggest mean granule diameter and highest SP respectively, exhibited the highest freeze‐thaw stability. Singhal and Kulkarni (1990) attributed smaller starch granule size to lower freeze‐thaw stability. The exudation of water from the starch gels is probably due to separation of the phases during ice crystal formation resulting in concentration of amylose and amylopectin in the unfrozen matrix and when heat is applied, expulsion of water from inter‐and intra‐ molecular associations occurs (Soni, Sharma, Srivasta, & Gharia, 1990).

Earlier findings have demonstrated low freeze‐thaw stability for plantain banana starch (Bello‐Perez et al., 1999) and white plantain starch (Nwokocha & Williams, 2009). The EAHB starch freezing–thawing results imply that these starches may not be appropriate for use in their native state in food systems involving refrigeration or freezing processes.

## CONFLICT OF INTEREST

We hereby declare that all the authors of this manuscript have no conflict of interest pertaining to this study whatsoever.

## References

[fsn3505-bib-0001] Anggraini, V. , Sudarmonowati, E. , Hartati, N. S. , Suurs, L. , & Visser, R. G. F. (2009). Characterization of cassava starch attributes of different genotypes. Starch/Stärke, 61, 472–481.

[fsn3505-bib-0002] AOAC . (1995) Official methods of analysis(16th edn). Washington, DC.

[fsn3505-bib-0003] Bello‐Perez, L. A. , Agama‐Acevedo, E. , Sanchez‐Hernandez, L. , & Paredes‐Lopez, O. (1999). Isolation and partial characterization of banana starches. Journal of Agriculture and Food Chemistry, 47, 854–857. 10.1021/jf980828t 10552380

[fsn3505-bib-0004] Bello‐Pérez, L. A. , de Léon, Y. P. , Agama‐Acevedo, E. , & Paredes‐López, O. (1998). Isolation and partial characterization of amaranth and banana starches. Starch/Stärke, 50(10), 409–413.

[fsn3505-bib-0005] Bello‐Pérez, L. A. , García‐Suárez, F. J. , Méndez‐Montealvo, G. , do Nascimento, O. J. R. , Lajolo, F. M. , & Cordenunsi, B. R. (2006). Isolation and characterization of starch from seeds of *Araucaria brasiliensis*: A novel starch for application in food industry. Starch/Stärke, 58, 283–291.

[fsn3505-bib-0006] Bello‐Pérez, L. A. , & Paredes‐López, O. (2009). Starches of some crops, changes during processing and their nutraceutical potential. Food Engineering Reviews, 1, 50–65.

[fsn3505-bib-0007] BeMiller, J. N . (2007). Carbohydrate chemistry for food scientists (2nd edn). St. Paul, MN: American Association of Cereal Chemists, Inc (AACC).

[fsn3505-bib-0009] Coulibaly, S. , Nemlin, J. G. , & Amani, G. N. (2006). Isolation and partial characterisation of native starches of new banana and plantain hybrids (*Musa* spp.) in comparison with that of plantain variety Orishele. Starch/Stärke, 58, 360–370.

[fsn3505-bib-0010] de la Torre‐Gutiérrez, L. , Chel‐Guerrero, Luis A. , & Betancur‐Ancona, D. (2008). Functional properties of square banana (*Musa balbisiana*) starch for square banana starch. Food Chemistry, 106, 1138–1144.

[fsn3505-bib-0011] Eggleston, G. , Swennen, R. , & Akoni, S. (1992). Physicochemical studies on starches isolated from plantain cultivars, plantain hybrids and cooking bananas. Starch/Stärke, 44, 121–128.

[fsn3505-bib-0012] Garcia, E. , & Lajolo, F. M. (1988). Starch transformation during banana ripening: The amylase and glucosidase behavior. Journal of Food Science, 53(4), 1181–1186.

[fsn3505-bib-0013] Goff, H. D. (2004). Modified starches and the stability of frozen foods In Eliasson A. C. (Ed.), Starch in food: Structure function and applications (pp. 425–440). Cambridge: Woodhead Publishing.

[fsn3505-bib-0014] Gold, C. S. , Bagabe, M. J. , & Ssendege, R. (1999). Banana weevil, *Cosmopolites sordidus* (Germar) (Coleoptera: Curculionidae) tests for suspected resistance to carbofuran and dieldrin in Masaka District Uganda, Africa. Entomology, 7, 189–196.

[fsn3505-bib-0015] Hoover, R. (2001). Composition, molecular structure, and physicochemical properties of tuber and root starches: A review. Carbohydrate Polymers, 45, 253–267.

[fsn3505-bib-0016] Hoover, R. , Sailaja, Y. , & Sosulski, F. (1996). Characterization of starches from wild and long grain brown rice. Food Research International, 29, 99–107.

[fsn3505-bib-0017] Huber, K. C. , & BeMiller, J. N. (2001). Location of sites of reaction within starch granules. Cereal Chemistry, 78, 173–180.

[fsn3505-bib-0018] International Association for Cereal Science and Technology (ICC) . (1995). Rapid pasting method using the newport rapid viscoanalyser. ICC Standard No. 162, International Association for Cereal Science and Technology.

[fsn3505-bib-0019] Kayisu, K. , Hood, L. F. , & Vansoest, P. J. (1981). Characterization of starch and fiber of banana fruit. Journal of Food Science, 46, 1885–1890.

[fsn3505-bib-0020] Moorthy, S. N. (2004). Tropical sources of starch In Eliasson A. C. (Ed.), Starch in food (pp. 321–359). New York: CRC Press.

[fsn3505-bib-0021] Muranga, F. I . (1998). Composition and physicochemical characteristics of starches of different banana varieties. PhD thesis. Makerere University, Kampala, Uganda.

[fsn3505-bib-0022] Nunez‐Santiago, M. C. , Bello‐Perez, L. A. , & Tecante, A. (2004). Swelling‐solubility characteristics, granule size distribution and rheological behavior of banana (*Musa paradisiaca*) starch. Carbohydrate Polymers, 56, 65–75.

[fsn3505-bib-0023] Nwokocha, L. M. , & Williams, P. A. (2009). Some properties of white and yellow plantain (*Musa paradisiaca*, Normalis) starches. Carbohydrate Polymers, 76, 133–138.

[fsn3505-bib-0024] Singhal, R. S. , & Kulkarni, P. R. (1990). Some properties of *Amaranthus paniculatas* (Rajgeera) starch pastes. Starch/Stärke, 42(1), 5–7.

[fsn3505-bib-0025] Soni, P. , Sharma, H. , Srivasta, H. , & Gharia, M . (1990). Physicochemical properties of *Canna edulis* starch‐Comparison with maize starch. Starch/Stärke, 42(12), 460–464.

[fsn3505-bib-0026] Srichuwong, S. , Sunarti, T. C. , Mishima, T. , Isono, N. , & Hisamatsu, M. (2005). Starches from different botanical sources. II. Contribution of starch structure to swelling and pasting properties. Carbohydrate Polymers, 62, 25–34.

[fsn3505-bib-0027] Tenywa, M. M. , Isabirye, M. I. , Lal, R. , Lufafa, A. , & Achan, P. (1999). Cultural practices and production constraints in smallholder banana‐based cropping systems of Uganda's Lake Victoria basin. Africa Crop Sci. J., 7(4), 613–623.

[fsn3505-bib-0028] Vatanasuchart, N. , Niyomwit, B. , & Wongkrajang, K. (2012). Resistant starch content, in vitro starch digestibility and physicochemical properties of flour and starch from Thai bananas. Maejo International Journal of Science and Technology, 6, 259–271.

[fsn3505-bib-0029] Waliszewski, K. N. , Aparicio, M. A. , Bello, L. A. , & Monroy, J. A. (2003). Changes of banana starch by chemical and physical modification. Carbohydrate Polymers, 52, 237–242.

[fsn3505-bib-0030] Wani, A. A. , Singh, P. , Shah, M. A. , Wani, I. A. , Götz, A. , Schott, M. , & Zacherl, C. (2013). Physico chemical, thermal and rheological properties of starches isolated from newly released rice cultivars grown in Indian temperate climates. LWT‐Food Science Technology, 53, 176–183.

[fsn3505-bib-0031] Waterschoot, J. , Gomand, S. V. , Fierens, E. , & Delcour, J. A. (2015). Production, structure, physicochemical and functional properties of maize, cassava, wheat, potato and rice starches. Starch/Stärke, 67, 14–29.

[fsn3505-bib-0032] Williams, P. C. , Kuzina, F. D. , & Hlynka, I. (1970). A rapid calorimetric procedure for estimating the amylose content of starches and flours. Cereal Chemistry, 47, 411–420.

[fsn3505-bib-0033] Zhang, P. , & Hamaker, B. R. (2012). Banana starch structure and digestibility. Carbohydrate Polymers, 87, 1552–1558.

[fsn3505-bib-0034] Zhang, P. , Whistler, R. L. , BeMiller, J. N. , & Hamaker, B. R. (2005). Banana starch: Production, physicochemical properties, and digestibility: A review. Carbohydrate Polymers, 59, 443–458.

